# Advanced alcoholic liver disease driven by a proferroptotic diet

**DOI:** 10.1016/j.jlr.2025.100898

**Published:** 2025-09-11

**Authors:** Yonggang Liang, Yanchao Xu, Megan Virostek, Ann Johnson, Bret Evers, Yaqin Deng, Yawen Meng, Jeffrey G. McDonald, Philipp E. Scherer, Shaojie Cui, Jin Ye

**Affiliations:** 1 Department of Molecular Genetics, University of Texas Southwestern Medical Center, Dallas, TX, USA; 2 Department of Thoracic Surgery, The Second Affiliated Hospital of Nanchang University, Nanchang, Jiangxi, China; 3 Touchstone Diabetes Center, University of Texas Southwestern Medical Center, Dallas, TX, USA; 4 Center for Human Nutrition, University of Texas Southwestern Medical Center, Dallas, TX, USA; 5 Department of Pathology, University of Texas Southwestern Medical Center, Dallas, TX, USA; 6 College of Biomedicine and Health, College of Life Science and Technology, Huazhong Agricultural University, Wuhan, Hubei, China

**Keywords:** dietary fat, fibrosis, fish oil, glycerolipids, ferroptosis, lipids/peroxidation, liver, nuclear receptor/SREBP, olive oil, triglycerides

## Abstract

Alcoholic liver disease (ALD) encompasses a spectrum of disorders, with advanced ALD—characterized by liver fibrosis—representing a severe stage with high mortality. The National Institute on Alcohol Abuse and Alcoholism ALD mouse model, a classical approach to studying ALD by delivering alcohol through the Lieber-DeCarli (LD) diet, typically does not progress to advanced ALD. We previously determined that ferroptosis causes hepatocellular injury in this model. Here, we speculate that the enrichment of MUFAs and vitamin E, which inhibit ferroptosis, and the lack of the proferroptotic nutrient iron in the LD diet may limit the progression of ALD by inhibiting ferroptosis. To test this hypothesis, we modified the LD diet to generate a proferroptotic LD (PFLD) diet by depleting vitamin E, increasing dietary levels of iron, and replacing MUFAs with PUFAs that drive ferroptosis. Upon feeding alcohol through the PFLD diet, ∼30% of the mice developed liver fibrosis and macrosteatosis, hallmarks of advanced ALD. These pathological changes were associated with exacerbated ferroptosis, possibly driven by overaccumulation of PUFA-containing triglycerides. Our findings underscore the critical role of dietary lipid composition in determining ALD severity, and demonstrate that feeding alcohol through the PFLD diet may serve as a mouse model for advanced ALD.

## Graphical Abstract

**Figure:**
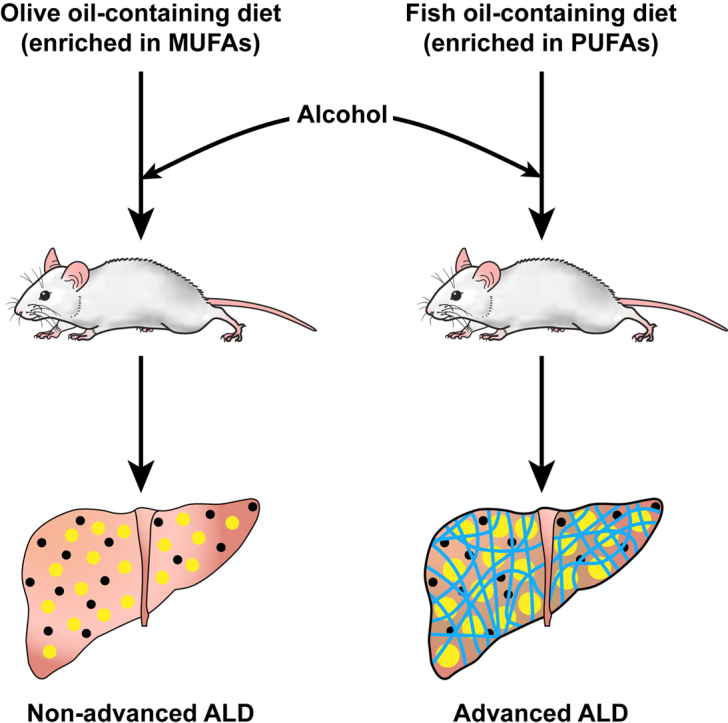

Alcoholic liver disease (ALD), which is caused by overconsumption of alcohol, is one of the most prevalent liver diseases in the United States (1, 2). ALD begins with alcoholic hepatosteatosis, a relatively benign disease characterized by accumulation of triglycerides (TGs) in hepatocytes accompanied by limited liver damage (2). A fraction of ALD patients progress further to alcoholic steatohepatitis (ASH), characterized by hepatic inflammation resulting from more severe hepatic damage (2). Severe ASH can lead to alcoholic hepatitis (AH), an acute clinical presentation of ALD, and alcoholic cirrhosis, a chronic end stage of ALD (2). Advanced ALD, which includes AH and alcoholic cirrhosis, is associated with high mortality and marked by the appearance of liver fibrosis (3). There is currently no effective treatment for advanced ALD.

A major obstacle in developing effective treatments for advanced ALD is the lack of an animal model for the disease. The National Institute on Alcohol Abuse and Alcoholism (NIAAA) model, the most extensively used mouse model of ALD, is based on feeding mice with the Lieber-DeCarli (LD) diet supplemented with alcohol for 10 days followed by a gavage of alcohol on day 11 (4). This model recapitulates early features of ASH but not advanced ALD (4, 5), thus limiting the application of the model to develop strategies to treat advanced ALD.

Using the NIAAA model, we recently reported that alcohol induces hepatic damage through ferroptosis (6), a cell death pathway triggered by iron-catalyzed peroxidation of PUFAs (7). Ferroptosis is caused by an accumulation of peroxidized PUFAs incorporated into phospholipids and is countered by glutathione peroxidase 4 (GPX4), which uses GSH as the reductant to reduce phospholipid peroxides (8). In addition to GPX4, ferroptosis is inhibited by certain nutrients, such as MUFAs, which compete with PUFAs for incorporation into phospholipids (9, 10, 11), and vitamin E, a lipid-soluble antioxidant that inhibits PUFA peroxidation (12).

In the current study, we showed that inhibition of ferroptosis by nutrients in the standard LD diet was likely the reason why this mouse model does not reach the stage of advanced ALD. By modifying the diet to promote ferroptosis, we observed that alcohol-fed mice developed features consistent with advanced ALD. Our results highlight the pivotal role of dietary fatty acid composition in driving the progression of ALD.

## Materials and methods

### Materials

We obtained LD control liquid diet, LD ethanol liquid diet, proferroptotic LD (PFLD) (vitamin E deficient, 150 mg/l iron, menhaden fish oil substituted for olive oil) liquid diet, and maltose dextrin from Bio-Serv; fatty acid-free BSA, isopropanol, chloroform, Weigert′s hematoxylin set, PBS with 0.05% Tween and Rabbit anti-actin from Sigma-Aldrich; Halt protease inhibitor cocktail, UltraPure™ 1 M Tris-HCl buffer, EDTA (0.5 M), UltraPure™ 10% SDS, NaCl (5 M), SYBR Green PCR Master Mix, Pierce BCA Protein Assay Kit, SuperSignal™ West Pico PLUS Chemiluminescent Substrate, Taqman reverse transcription reagents, and DNA-free™ DNA Removal Kit from Thermo Fisher Scientific; Picrosirius Red Stain Kit from Polysciences; 10% NP-40, GSH/GSSG Ratio Detection Assay Kit II (Abcam), Iron Assay Kit, and Deproteinizing Sample Preparation Kit from Abcam; Bluing Reagent and Gills II hematoxylin from Richard-Allan Scientific; 32% paraformaldehyde aqueous solution and xylene from Fisher Scientific; ethyl alcohol (100%) from Greenfield Global USA, Inc; Rabbit anti-Calnexin from Enzo Life Sciences; and Peroxidase AffiniPure Goat Anti-Rabbit IgG (H+L) from Jackson ImmunoResearch. Rabbit monoclonal antibodies IgG-20B12 and IgG-5H7c were generated by our department and reported previously (13, 14).

### Animal studies

All animal experiments described in this work were approved and conducted under the oversight of the University of Texas Southwestern Institutional Animal Care and Use Committee. All mice were housed in colony cages in a room with a 12-h light/12-h dark cycle. Alcohol feeding was conducted on female C57BL/6 mice aged from 12 to 20 weeks, in accordance with established protocols (4). Briefly, mice were fed with the LD diet (Bio-Serv; product no.: F1259SP) for 5 days to acclimatize them to liquid diets. Afterward, on day 0, they were fed with the LD or PFLD diet (custom-made by Bio-Serv; product no.: F10527SP) supplemented with 5% ethanol or an isocaloric amount of maltose dextrin as previously described (4). On day 11 and day 19 in the experiments shown in 1, 2, 3, 4, 5, respectively, alcohol-fed and pair-fed mice received a single dose of alcohol (5 g/kg body weight) and isocaloric maltose dextrin, respectively, by oral gavage. Mice were euthanized by isoflurane 9 h after the gavage.

**Fig. 1:**
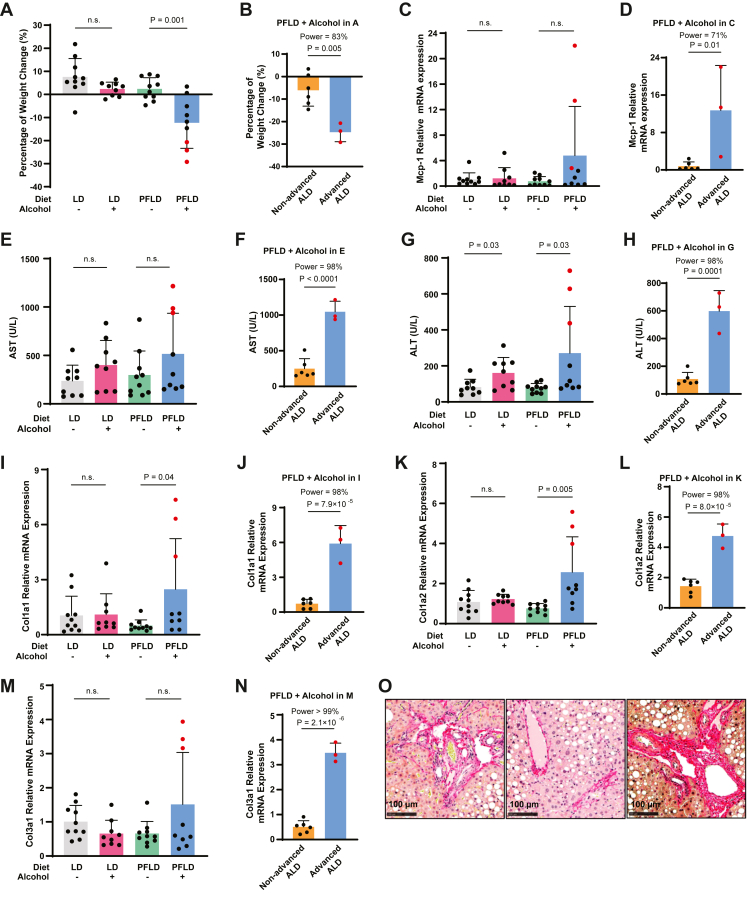
Mice fed with alcohol through the PFLD develop advanced ALD. A and B: Percentage of weight change in mice fed with or without alcohol through the indicated diet (A). The same data were used for statistical analysis comparing weight changes between mice with and without advanced ALD among those fed with alcohol through the PFLD diet (B). C and D, I–N: Relative expression levels of Mcp-1 (C), Col1a1 (I), Col1a2 (K), and Col3a1 (M) mRNA in livers of the mice fed with or without alcohol through the indicated diet were measured by quantitative RT-PCR, with the average value from the mice fed with the LD diet in the absence of alcohol set at 1. The same data from the preceding panel were used for statistical analysis comparing levels of these mRNA between mice with and without advanced ALD among those fed with alcohol through the PFLD diet (D, J, L, and N). E–H: Serum levels of aspartate transaminase (E) and alanine transaminase (G) in mice fed with or without alcohol through the indicated diet. The same data were used for statistical analysis comparing aspartate transaminase (F) and alanine transaminase (H) values between mice with and without advanced ALD among those fed with alcohol through the PFLD diet. O: Representative Picrosirius Red-stained liver sections from mice with advanced ALD. A–N: Data from the mice with advanced ALD are highlighted in red. Data are reported as mean ± SD. Statistical analysis was performed through unpaired, two-tailed *t*-tests, with *P* values and power values derived from the *P* values displayed on indicated comparisons.

**Fig. 2:**
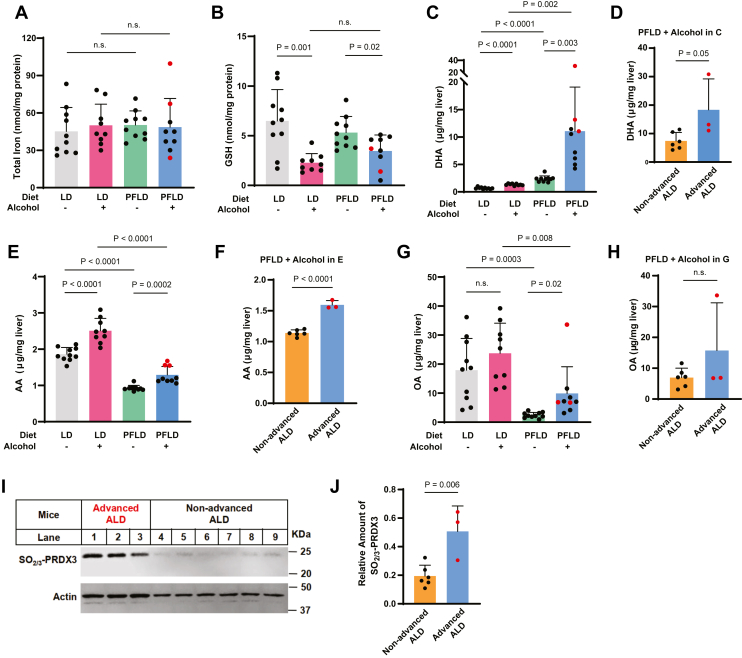
Mice with advanced ALD contain higher levels of PUFAs in livers (A–C, E, and G). The hepatic levels of iron (A), GSH (B), DHA (C), AA (E), and OA (G) in mice fed with or without alcohol through the indicated diet were determined as described in the Materials and Methods section. D, F, and H: The same data from the preceding panel were used for statistical analysis comparing levels of the fatty acid in livers between mice with and without advanced ALD among those fed with alcohol through the PFLD diet. I: Immunoblot analysis of livers from the mice shown in (D) with the indicated antibody. J: Quantification of immunoblot signals of hyperoxidized PRDX3 normalized to that of actin shown in I. A–H and J: Data from the mice with advanced ALD are highlighted in red. Data are reported as mean ± SD. Statistical analysis was performed through unpaired, two-tailed *t*-tests.

**Fig. 3:**
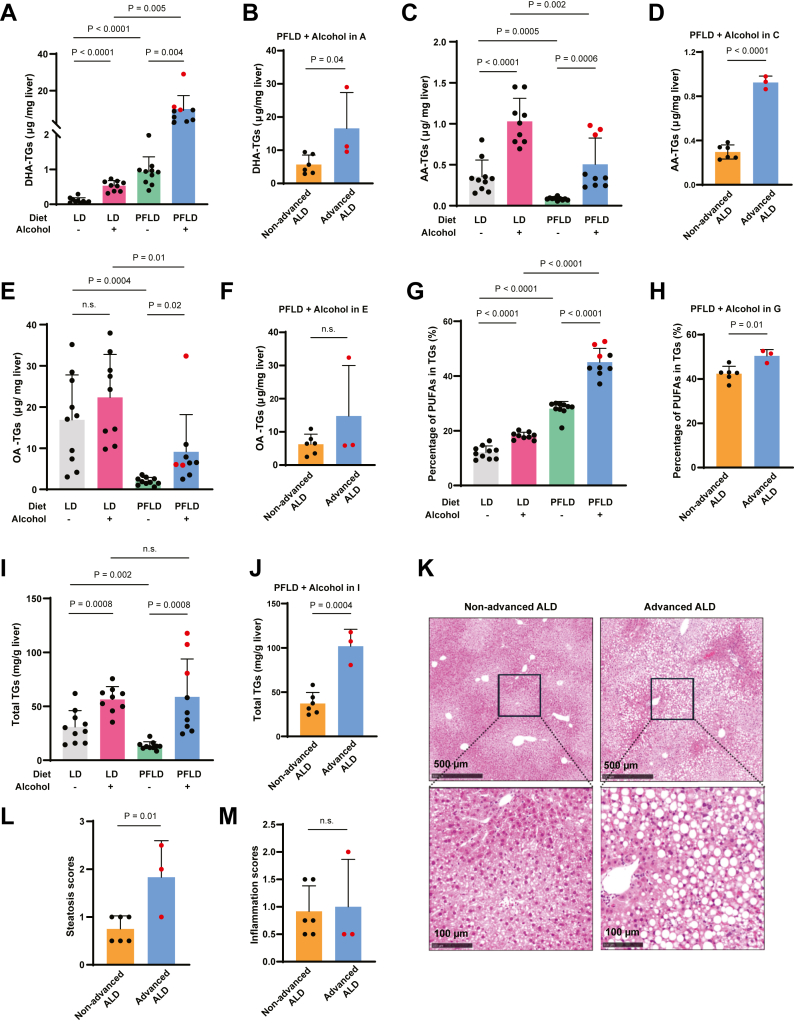
Accumulation of PUFA-TGs in mice with advanced ALD (A, C, E, G, and I). The hepatic levels of DHA-TGs (A), AA-TGs (C), OA-TGs (E), the percentage of polyunsaturated TGs (G), and total TGs (I) in mice fed with or without alcohol through the indicated diet were determined as described in the Materials and methods section. B, D, F, H, and J: The same data from the preceding panel were used for statistical analysis comparing levels of the indicated TGs in livers between mice with and without advanced ALD among those fed with alcohol through the PFLD diet. K: Representative H&E-stained liver sections of the mice fed with alcohol through the PFLD with or without advanced ALD. L and M: Statistical analysis comparing steatosis (L) and inflammation scores (M) of livers between mice with and without advanced ALD among those fed with alcohol through the PFLD diet. A–J, L, and M: Data from the mice with advanced ALD are highlighted in red. Data are reported as mean ± SD. Statistical analysis was performed through unpaired, two-tailed *t*-tests.

**Fig. 4:**
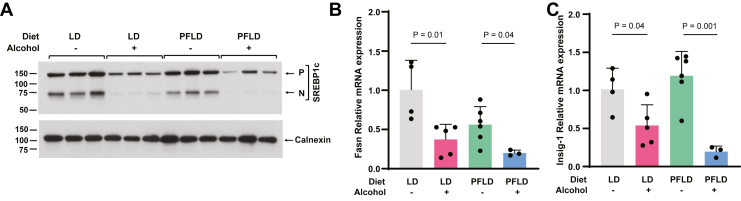
Alcohol induces hepatic TG accumulation independent of SREBP1c. A: The level of SREBP1c in livers of the mice fed with or without alcohol through the indicated diet was determined by immunoblot analysis. P and N denote the precursor and the cleaved, active nuclear form of SREBP1c, respectively. B and C: The amount of fatty acid synthase (Fasn) (B) and insulin-induced gene 1 (Insig-1) (C) mRNA in livers of the mice was determined by quantitative RT-PCR as described in Figure 1C.

**Fig. 5:**
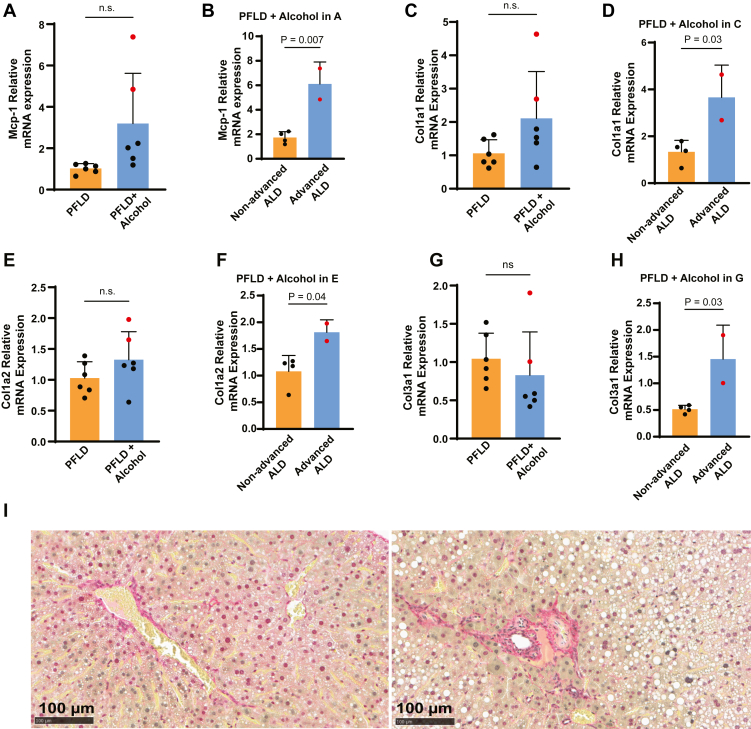
Prolonging alcohol feeding fails to increase the percentage of mice with advanced ALD. A–H: Measurement of Mcp-1, Col1a1, Col1a2, and Col3a1 mRNA was performed as described in Figures 1C–D, I–J, K–L and M–N, respectively, with the average value from the mice fed with the PFLD diet in the absence of alcohol set at 1. Data from the mice with advanced ALD are highlighted in red. Statistical analysis was performed through unpaired, two-tailed *t*-tests. I: Representative Picrosirius Red-stained liver sections from mice with advanced ALD.

### Quantitative RT-PCR

Total RNA was prepared from mouse livers with an RNA STAT-60 kit (Tel-Test, Inc) and was treated with a DNA-free™ DNA Removal Kit (Thermo Fisher Scientific). RNA (2 μg) of each sample was reverse transcribed with a complementary DNA reverse transcription kit (Thermo Fisher Scientific) and subjected to quantitative PCR by using SYBR Green PCR Master Mix (Thermo Fisher Scientific). All reactions were carried out in triplicate, and the relative amount of all mRNAs was calculated using the comparative threshold cycle (C_T_) method. The mRNA levels of mouse cyclophilin were used as the invariant control for mouse liver samples.

### Immunoblot analysis

Livers were homogenized in buffer A (50 mM Tris-HCl, 150 mM NaCl, 1% NP-40, 0.1% SDS, 0.5% sodium deoxycholate, 1 mM EDTA, and protease inhibitor cocktail). Aliquots of the lysate (10 μg protein) were subjected to 13% SDS-PAGE followed by immunoblot analysis with IgG-20B12 (13) (1 μg/ml) to detect sterol regulatory element binding protein 1c (SREBP1c), 5H7c (14) (0.1 μg/ml) to detect hyperoxidized PRDX3, anti-Calnexin (1:20,000 dilution) to detect calnexin, and anti-actin (1:20,000 dilution) to detect actin. These primary antibodies were diluted in PBS with 0.05% Tween buffer containing 5% BSA. Bound antibodies were visualized with a peroxidase-conjugated secondary antibody (1:5,000 dilution) using the Super Signal ECL-HRP substrate system. The immunoblot signals were captured through Li-COR (Image Studio™, version 5.0) (Fig. 2I) or detected after exposure to CL-XPosure™ Films (Fig. 4A).

### Iron assay

Hepatic total iron was measured by the Iron Assay kit (Abcam) according to the manufacturer’s protocol.

### GSH assay

Hepatic GSH levels were assessed by GSH/GSSG Ratio Detection Assay Kit II according to the manufacturer’s protocol.

### Lipid analysis

Fatty acids incorporated into TGs and phospholipids were measured as described previously (15). TGs and phospholipids were extracted from livers and separated using a three-phase liquid extraction method. Following saponification, fatty acids released from TGs and phospholipids were derivatized with triethylamine and pentafluorobenzyl bromide and analyzed through GC/MS by electron capture negative ionization. The data were processed using the MassHunter software (Agilent). The amount of total fatty acids in livers was calculated by addition of that in TGs and phospholipids. The amount of total TGs in livers was measured by the University of Texas Southwestern Medical Center Metabolic Phenotyping Core.

### Pathological analysis

Livers fixed with 4% paraformaldehyde for 16 h were switched to 70% ethanol for long-term storage. Following delipidation and dehydration using the Tissue-Tek Vip 6 Processor, livers were embedded in paraffin, sectioned at 5 μm, deparaffinized with xylene, and dehydrated with ethanol at gradient concentrations. For H&E staining, sections were stained with Gills II hematoxylin (Richard-Allan Scientific; catalog no.: 7211) for 5 min, treated with Bluing Reagent (Richard-Allan Scientific; catalog no.: 7301) for 30 s, and stained with eosin for 4 min. For Picrosirius Red staining, sections were stained in Weigert’s hematoxylin (Sigma; catalog no.: HT1079-1SET) for 20 min, followed by staining according to the instruction of the Picrosirius Red Stain Kit (Polysciences; catalog no.: 24901). The sections were scanned by Nanozoomer S60 (University of Texas Southwestern Medical Center Whole Brain Microscopy Facility, Research Resource Identifier: SCR_017949) and viewed using the NDP.View2 software.

Steatosis and portal tract inflammation were scored on a 4-point scale (0—none, 1—mild, 2—moderate, and 3—severe) using a scheme modified from a prior study (16). The average scores were calculated from multiple liver sections per mouse by a board-certified pathologist.

## Results

### The PFLD diet enables mice to develop advanced ALD

Previous studies demonstrated that feeding mice with alcohol through the NIAAA protocol for 11 days led to phenotypes mimicking early stages of ASH, but prolonging the alcohol treatment through the same protocol reduced the severity of ALD in mice (5). A possible explanation for this observation is that the LD diet on which the NIAAA model is based may contain nutrients that alleviate hepatic damage upon long-term feeding. Inasmuch as alcohol induces hepatic damage in this model through ferroptosis (6), we investigated whether the LD diet is compatible with ferroptosis. This analysis revealed that the LD diet is based on olive oil (Table 1) that is highly enriched in MUFAs, particularly oleic acid (OA) (17), a potent inhibitor of ferroptosis (9, 10, 11). In addition, the LD diet contains vitamin E, another ferroptosis inhibitor (7), but the amount of iron, a critical element that drives ferroptosis (7), is ∼5 times lower than that of the standard chow diet (Table 1). Thus, the LD diet might counter the ferroptotic stress induced by alcohol, thereby reducing the severity of hepatic damage preventing the development of advanced ALD.

**Table 1:** Comparison between the LD and PFLD diets

Diet	LD	PFLD
Olive oil (g/l)	28.4	0
Menhaden fish oil (g/l)	0	28.4
Iron (mg/l)	9.3	150
Vitamin E (IU/l)	39.3	0

Generation of the PFLD diet through alteration of the indicated nutrients in the LD diet.

To test this hypothesis, we modified the LD diet by replacing olive oil with fish oil (Table 1), which is enriched in PUFAs that promote ferroptosis, and was shown previously to exacerbate alcohol-induced liver damage in rodents (7, 18). We also removed vitamin E from the diet and increased the amount of iron to a level that is three times as much as that in the chow diet (Table 1), a dose previously shown to be well tolerated in mice without inducing ferroptosis in the liver (19). We then fed mice with this new diet, which we designated as the PFLD diet, with or without alcohol according to the NIAAA protocol, and performed a side-by-side comparison with the original NIAAA model in which mice were fed with alcohol through the original LD diet. As previously reported (4), feeding alcohol through the LD diet did not prevent mice from gaining body weight (Fig. 1A). In contrast, while mice fed with the PFLD diet alone gained weight, those fed with the PFLD diet supplemented with alcohol lost their body weight (Fig. 1A). Interestingly, their weight loss showed a bimodal distribution, as ∼30% of them displayed more severe weight loss (Fig. 1A highlighted in red) than the rest of the mice subject to the same treatment (Fig. 1B). Inasmuch as severe weight loss is a common feature for AH (20), we measured hepatic expression of *Mcp-1*, a marker for AH (21, 22). Feeding alcohol through the PFLD diet, when analyzed as a single group, did not significantly elevate the *Mcp-1* expression level (Fig. 1C). However, if stratified according to the bimodal distribution of the weight loss, those 30% of the mice with more severe weight loss did show higher expression of the gene (Fig. 1D). Consistent with these observations, their levels of aspartate transaminase and alanine transaminase, markers for liver damage, were markedly higher than the rest of the mice (Fig. 1E–H). These observations suggest that ∼30% of the mice fed with alcohol through the PFLD diet might progress to advanced ALD resembling AH.

We then examined liver fibrosis, a hallmark of advanced ALD (3). We first determined the expression of genes encoding collagen fibers that cause liver fibrosis. Feeding mice with alcohol through the PFLD but not the LD diet increased expression of *Col1a1* in livers (Fig. 1I). This increase was primarily contributed by the mice with more severe weight loss, as the expression level of the gene was significantly higher in these mice than the rest of the mice subject to the same treatment (Fig. 1J). Expression of *Col1a2* and *Col3a1* followed the same trend (Fig. 1K–N). Indeed, among all the mice we tested, only those with more severe weight loss expressed higher levels of all three components of the collagen fibers (Fig. 1I–N). Consistent with gene expression results, Picrosirius Red staining showed the early deposition of collagen fibers around the portal tracts of their livers (Fig. 1O). These results demonstrated the presence of liver fibrosis in these mice, further supporting that they developed advanced ALD.

The results shown above revealed that mice fed with alcohol through the PFLD diet showed bimodal data distribution, as the values reflecting liver damage were much higher in mice with advanced ALD than the rest of the mice within the same feeding group, and this difference was not only statistically significant but also carried enough significant power (Fig. 1F, H, J, L, and N). The bimodal data distribution pattern made it difficult to compare mice fed with alcohol through the PFLD diet with other feeding groups, which did not show such a pattern, through conventional statistical analyses. Thus, in the rest of the study, in addition to comparing results from different feeding groups, we also compared data from the mice with or without advanced ALD within the same feeding group to demonstrate the difference among them and the existence of the bimodal data distribution pattern.

### Mice with advanced ALD accumulate more PUFAs in their livers

The results shown above suggest that feeding alcohol through the PFLD diet, which is more favorable to ferroptosis, led to the development of advanced ALD in ∼30% of the mice. Thus, ferroptotic stresses imposed by the PFLD diet might exceed the threshold required for hepatocytes to successfully defend against ferroptotic attacks launched by alcohol. A possible stress might be higher levels of iron in the PFLD diet. However, the amounts of hepatic iron in the mice fed with the PFLD diet, regardless of alcohol feeding, were not significantly higher than that in the mice fed with the LD diet (Fig. 2A). Another possible stress might be depletion of vitamin E in the PFLD diet. In addition to inhibiting lipid peroxidation, vitamin E is also required to maintain adequate hepatic levels of GSH (23, 24), a cofactor essential for GPX4-catalyzed detoxification reaction that protects cells from ferroptosis (7). However, while alcohol feeding reduced levels of GSH, this reduction was not further amplified by the PFLD diet (Fig. 2B). We then determined whether replacing MUFAs with PUFAs was responsible for the PFLD diet to facilitate the development of advanced ALD. Feeding mice with the PFLD diet elevated hepatic levels of DHA, the most abundant ω-3 PUFA in livers, presumably because DHA is the major component of fish oil present in the PFLD diet (Fig. 2C). Alcohol feeding increased the hepatic levels of DHA, and this increase was further amplified when alcohol was administered through the PFLD diet (Fig. 2C). Among these mice, the hepatic levels of DHA were significantly higher in mice with advanced ALD (Fig. 2D). Unlike DHA, the amounts of arachidonic acid (AA), the most abundant ω-6 PUFA in livers, were lower in livers of the mice fed with the PFLD diet, presumably because fish oil is not enriched in ω-6 PUFAs, whereas olive oil in the LD diet contains a significant amount of linoleic acid that can be converted to AA in livers (Fig. 2E). Despite this difference, alcohol feeding still increased hepatic levels of AA (Fig. 2E), and the amounts of AA in livers of the mice with advanced ALD were significantly higher than that of the mice subject to the same treatment but without advanced ALD (Fig. 2F). These observations suggest that alcohol consumption elevates hepatic PUFA levels, and this increase is exaggerated when alcohol is fed through a diet enriched in PUFAs. In this study, the PUFA that was particularly elevated was DHA, the major component of fish oil present in the PFLD diet.

In contrast to PUFAs that drive ferroptosis, OA, a MUFA that inhibits ferroptosis, accumulated in the livers of the mice fed with the LD diet, as OA is the major component of olive oil present in this diet (Fig. 2G). While alcohol feeding increased the amounts of OA in livers of the mice fed with the PFLD diet, their levels were still significantly lower than that of the mice fed with the LD diet (Fig. 2G). The hepatic levels of OA were also not particularly elevated in mice with advanced ALD (Fig. 2G, H).

The results shown above demonstrated that livers of the mice with advanced ALD accumulated higher levels of PUFAs, particularly DHA, but not MUFAs such as OA. Considering that PUFAs and MUFAs drive and inhibit ferroptosis, respectively, these observations suggest that hepatocytes in mice with advanced ALD might be subject to more severe ferroptosis. Indeed, immunoblot analysis revealed that levels of hyperoxidized PRDX3, a ferroptosis marker (6), were higher in mice with advanced ALD than those subject to the same alcohol feeding procedure but without advanced ALD (Fig. 2I, J). Thus, exacerbated ferroptosis of hepatocytes appeared to contribute to the development of advanced ALD.

### Mice with advanced ALD develop macrosteatosis driven by PUFA-TG accumulation

The alcohol-induced hepatic accumulation of PUFAs in mice, particularly those fed with the PFLD diet, may lead to increased synthesis of PUFA-containing TGs. Indeed, the amounts of DHA-containing TGs followed that of DHA in livers: Alcohol feeding increased the hepatic levels of DHA-containing TGs, and this increase was further exaggerated when alcohol was administered through the PFLD diet (Fig. 3A). Among the mice subject to this treatment, the hepatic levels of DHA-containing TGs were higher in mice with advanced ALD (Fig. 3B). Similarly, alcohol feeding elevated hepatic levels of AA-containing TGs (Fig. 3C), and the amounts of AA-containing TGs were higher in mice with advanced ALD than in those subject to the same treatment but without the disease (Fig. 3D). The amounts of OA-containing TGs also followed that of OA in livers, as their hepatic levels were lower in the mice fed with the PFLD diet (Fig. 3E) and were not elevated in mice with advanced ALD (Fig. 3F). Consistent with these observations, alcohol feeding elevated the percentage of PUFA-containing TGs in livers, and this elevation was much more prominent in mice fed with the PFLD diet (Fig. 3G). Remarkably, this percentage reached the highest levels in mice with advanced ALD (Fig. 3G). Their PUFA-TG percentage was significantly higher than that of the mice subject to the same alcohol feeding procedure but without advanced ALD (Fig. 3H).

We subsequently measured total TGs in the livers. Similar to the previous report (5), feeding mice with alcohol through the LD diet increased hepatic TG levels (Fig. 3I). Feeding mice with the PFLD diet in the absence of alcohol markedly reduced hepatic TG levels (Fig. 3I), as PUFAs enriched in fish oil present in this diet inhibit synthesis of fatty acids by inactivating SREBP1c (25), a transcription factor that activates all genes involved in fatty acid synthesis (26). Remarkably, alcohol feeding markedly increased the amounts of TGs in the livers of the mice fed with the PFLD diet so that their hepatic TG levels were similar to that of the mice fed with alcohol through the LD diet (Fig. 3I). This increase was primarily driven by the mice with advanced ALD, as their hepatic TG levels were more than 2-fold higher than that of the mice subject to the same treatment but without advanced ALD (Fig. 3J). Consistent with these observations, pathological analysis revealed that mice with advanced ALD displayed macrosteatosis, whereas those fed with alcohol through the same procedure but without advanced ALD primarily showed microsteatosis (Fig. 3K). Among these mice, those with advanced ALD had higher steatosis but not inflammation scores (Fig. 3L, M). These results suggest that higher levels of steatosis are correlated with advanced ALD, as this pathological feature may reflect overaccumulation of PUFA-TGs in livers that could lead to excessive ferroptosis.

### ALD induces hepatic TG accumulation through a mechanism different from that of nonalcoholic fatty liver disease

In mouse models of nonalcoholic fatty liver disease (NAFLD), TG accumulation is primarily driven by endogenous synthesis of fatty acids as a result of proteolytic activation of SREBP1c (27). We thus investigated whether the same pathway is responsible for ethanol-induced TG accumulation in livers. Inasmuch as SREBP1c activation is very sensitive to feeding status (28), we selected mice that remained fasted following the gavage for this analysis. These mice should be considered as being fasted for 9 h, a condition that inhibits SREBP1c activation. Judging by the levels of the cleaved active nuclear form of SREBP1c, PUFAs enriched in the PFLD diet did not further inhibit SREBP1c under this condition (Fig. 4A). In contrast, alcohol feeding, regardless of the diet, still inhibited SREBP1c under this circumstance (Fig. 4A), leading to reduced expression of known SREBP1c target genes, such as fatty acid synthase (*Fasn*) and insulin-induced gene 1 (*Insig-1*) (26) (Fig. 4B, C). Thus, in contrast to NAFLD, alcohol appears to induce hepatic TG accumulation through a mechanism independent of SREBP1c activation.

### Prolonging alcohol feeding through the PFLD diet does not increase the percentage of mice that develop advanced ALD

Considering that only ∼30% of the mice developed advanced ALD upon feeding alcohol through the PFLD diet using the feeding schedule established by the NIAAA model, we wondered whether prolonging alcohol feeding would increase this percentage. To test this hypothesis, we set up an experiment similar to that shown in Figure 1 except that the mice were fed with the PFLD diet in the presence or absence of alcohol for up to 18 days before receiving a gavage of alcohol or maltose dextrin as a control on the next day. We had to sacrifice one mouse fed with alcohol for only 8 days in this experiment owing to the severe weight loss that made the mouse unlikely to survive further alcohol feeding. Amazingly, we still observed ∼30% of the mice, including the one that had been sacrificed before the termination of the experiment, developed advanced ALD judging by their expression of *Mcp-1* (Fig. 5A, B), *Col1a1* (Fig. 5C, D), *Col1a2* (Fig. 5E, F), and *Col3a1* (Fig. 5G, H), and the initiation of liver fibrosis surrounding the portal tracts revealed by Picrosirius Red staining (Fig. 5I). Thus, increasing alcohol feeding time did not increase the percentage of mice that developed advanced ALD.

## Discussion

The current study demonstrates that feeding mice with alcohol through the PFLD diet enabled them to develop advanced ALD. In contrast, the same alcohol feeding protocol based on the LD diet never caused advanced ALD in mice. Compared with the PFLD diet, the LD diet is enriched in OA and vitamin E that inhibit ferroptosis but lacks PUFAs and iron that drive ferroptosis. Considering that ferroptosis is responsible for alcohol-induced hepatic damage (6), the LD diet may reduce the severity of ferroptosis induced by alcohol, thereby preventing mice from developing advanced ALD. This conclusion was supported by the observation that mice fed with the LD diet accumulated high levels of OA, a potent inhibitor of ferroptosis (9, 10, 11). Thus, olive oil, the source of OA in the LD diet, may alleviate the severity of ALD. Considering that olive oil is the cornerstone of the Mediterranean diet (29), our finding might help to explain why alcohol consumption, a characteristic of the Mediterranean lifestyle, does not affect the overall health benefit of the diet. The protective effects of olive oil on advanced ALD were further demonstrated by our finding that substitution of olive oil with PUFA-enriched fish oil in the PFLD diet enabled mice to develop advanced ALD. In contrast to MUFAs such as OA, PUFAs stimulate ferroptosis by serving as substrates for lipid peroxidation (19). We discovered that alcohol, regardless of the diet, selectively caused PUFA accumulation in the liver. The PFLD diet, which is enriched in PUFAs, further exaggerated this accumulation. Under this circumstance, mice with the highest hepatic levels of PUFAs suffered from excess ferroptosis in their livers, leading to the development of advanced ALD marked by liver fibrosis. Our findings suggest the existence of a previously unrecognized deleterious interaction between alcohol and fish oil, which is frequently used to manage hypertriglyceridemia (30).

An important finding of the current study is that alcohol-induced PUFA accumulation contributes to hepatosteatosis, as alcohol, regardless of the diet, increased PUFA-containing TGs in livers. Our findings suggest that higher PUFA-TG levels are correlated with the severity of ALD, as mice with advanced ALD contained the highest hepatic levels of total TGs, as well as the highest percentage of PUFA-TGs. This conclusion is consistent with the pathological analysis showing that mice with advanced ALD displayed macrosteatosis and exhibited higher steatosis scores. Previous studies suggest that storing PUFAs in TGs is a mechanism to protect cells from ferroptosis, as this reaction sequesters PUFAs away from phospholipids, thus limiting phospholipid peroxidation (31, 32, 33, 34). However, it should be emphasized that ferroptosis in these studies is caused by artificial compounds that inhibit GPX4 (31, 34), which removes peroxidized phospholipids (8). These observations do not exclude the possibility that PUFA-TG peroxidation may also lead to ferroptosis, as reactions preventing PUFA-TG peroxidation may still be intact when ferroptosis was caused by deficiency in GPX4. Indeed, PUFA-TG peroxidation was associated with NAFLD (35), a disorder also caused by ferroptosis of hepatocytes (6). Thus, alcohol-induced accumulation of PUFA-TGs may contribute to ALD by providing substrates for lipid peroxidation that causes ferroptosis.

Exactly how alcohol leads to TG accumulation in livers remains unclear. In various mouse models of NAFLD, SREBP1c-activated lipogenic reactions are critical for lipid accumulation in livers (27). However, this mechanism does not appear to play an important role in lipid accumulation caused by ALD, as, rather than activating, alcohol actually inhibited proteolytic activation of SREBP1c. This observation is consistent with previous findings that alcohol feeding inhibited expression of lipogenic genes driven by SREBP1c (36, 37). Thus, although both diseases are marked by steatosis and are caused by ferroptosis (6), the lipid accumulation mechanism appears to be different between ALD and NAFLD. Considering recent reports that alcohol-induced hepatosteatosis is caused by lipolysis of adipocytes (36, 37), it is likely that PUFAs released from adipose tissues through lipolysis may contribute to alcohol-induced hepatic accumulation of PUFA-TGs. In this regard, it is interesting to note that the genetic variation of patatin-like phospholipase domain-containing protein 3 (PNPLA3[I148M]) is the most important genetic determinant of the risk of advanced ALD (38, 39, 40). PNPLA3 is a TG lipase with substrate specificity tailored to polyunsaturated TGs (41, 42). The I148M mutation markedly stabilizes PNPLA3, leading to increased lipase activity even though the mutation by itself reduced the enzymatic activity (43, 44). Considering that adipocytes express high levels of PNPLA3 (45), it will be interesting to investigate the role of adipose PNPLA3 on alcohol-induced hepatic PUFA accumulation.

A limitation of this study is that only ∼30% of mice fed with alcohol through the PFLD diet developed advanced ALD. Interestingly, most of the data obtained from mice subject to this treatment showed bimodal but not continuous distribution, with those who had advanced ALD at one extreme and the rest of the mice at another extreme. This data distribution pattern made it difficult to compare this group of mice as a whole to other treatment groups, as most statistical analyses assume continuous data distribution. Instead of this comparison, we demonstrated that within the group of mice fed with alcohol through the PFLD diet, the difference between values obtained from the mice with and without advanced ALD not only was statistically significant but also was carrying significant statistical power. While we do not understand why only a fraction of the mice developed advanced ALD under this experimental condition, this finding is consistent with the observation that only up to 20% of humans consuming excess alcohol develop advanced ALD. It will be interesting to determine whether this percentage could be increased by feeding mice carrying a mutant allele such as PNPLA3(I148M) that increases the risk of advanced ALD.

## Data availability

All data are contained in the article. Any additional information required to reanalyze the data reported in this article is available from JY upon request.

## Conflict of interest

The authors declare that they have no conflicts of interest with the contents of this article.
